# Pool Chemical–Associated Health Events in Public and Residential Settings — United States, 2003–2012, and Minnesota, 2013

**Published:** 2014-05-16

**Authors:** Michele C. Hlavsa, Trisha J. Robinson, Sarah A. Collier, Michael J. Beach

**Affiliations:** 1 Division of Foodborne, Waterborne, and Environmental Diseases, National Center for Emerging and Zoonotic Infectious Diseases, CDC; 2 Minnesota Department of Health

Pool chemicals are added to treated recreational water venues (e.g., pools, hot tubs/spas, and interactive fountains) primarily to protect public health by inactivating pathogens and maximizing the effectiveness of disinfection by controlling pH. However, pool chemicals also can cause injuries when handled or stored improperly. To estimate the number of emergency department (ED) visits for injuries associated with pool chemicals in the United States per year during 2003–2012, CDC analyzed data from the U.S. Consumer Product Safety Commission’s National Electronic Injury Surveillance System (NEISS). This report summarizes the results of that analysis. In 2012 alone, an estimated 4,876 persons (95% confidence interval [CI] = 2,821–6,930) visited an ED for injuries associated with pool chemicals. Almost half of the patients were aged <18 years. This report also describes a pool chemical–associated health event that occurred in Minnesota in 2013, which sent seven children and one adult to an ED. An investigation by the Minnesota Department of Health (MDH) determined the cause to be poor monitoring of or response to pool chemistry. Pool chemical–associated health events are preventable. CDC’s Model Aquatic Health Code (MAHC) (1) is a resource that state and local agencies can use to optimize prevention of injuries and illnesses associated with public treated recreational water venues, including pool chemical–associated health events.

NEISS captures data on ED visits for injuries associated with consumer products, including product codes (e.g., pool chemical code: 938); the most severe diagnosis; the most seriously injured body part; incident location; disposition, age, sex, and race/ethnicity of the patient; and two 71-character narrative fields to describe events leading to injury. These data are collected from a nationally representative probability sample of approximately 100 hospitals across the United States, and thus can be used to calculate national estimates. Each case was weighted based on the inverse probability of the hospital being selected, and the weights were summed to produce national estimates; 95% CIs were calculated, accounting for the sample weights and complex sampling design. Rates per 100,000 person-years were calculated using these estimates and U.S. Census Bureau population estimates (2).

In the United States during 2003–2012, the median estimated number of persons visiting an ED for pool chemical–associated injuries per year was 4,247 (range = 3,151–5,216) (Figure). In 2012, an estimated 4,876 persons (95% CI = 2,821–6,930; 1.6 per 100,000 person-years) visited an ED for injuries associated with pool chemicals (Table). Almost half (46.9%) of the patients were aged <18 years (an estimated 2,289 persons [95% CI = 965–3,613]; 3.1 per 100,000 person-years). The most frequent diagnosis was poisoning (an estimated 2,167 injuries [95% CI = 1,219–3,116]; 0.7 per 100,000 person-years). Of the 50 actual visits to NEISS-participating EDs resulting in a poisoning diagnosis, 46 (92.0%) stemmed from inhalation of vapors, fumes, or gases rather than ingestion. More than a third (36.1%) of the injuries occurred at a residence. Of the total 109 actual visits to NEISS-participating EDs, 79 (72.5%) occurred over the summer swim season (Saturday of Memorial Day weekend through Labor Day); 47 (43.1%) occurred on a Saturday or Sunday. No deaths were documented. Patients were injured when handling pool chemicals without using personal protective equipment such as goggles (especially while opening containers), when pool chemicals were added to the water just before the patient entered the water (frequently in residential and hotel settings), and when pool chemicals were not secured away from children.

In December 2013, a mother notified MDH that multiple persons had developed rashes and symptoms of respiratory illness after attending a child’s birthday party on the previous Saturday in December at an indoor hotel swimming pool and spa. MDH conducted a cohort study and enrolled all 12 party attendees, who were interviewed by telephone using a standardized questionnaire. Eight of the 12 reported developing a raised, red rash all over their body. Ill persons also reported headache, cough, sore throat, vomiting, and difficulty urinating. The eight ill persons reported illness onset 5.5–7.0 hours after first exposure to the swimming pool or spa. All eight ill persons sought medical attention at an ED, where their signs and symptoms were clinically diagnosed as chemical burns. Inspection by an MDH environmental health specialist 2 days after the birthday party revealed free chlorine* levels ≥15–30 ppm in both the swimming pool and spa, exceeding the state limit of 5.0 ppm. The pH was measured at 9.0 in both bodies of water, exceeding the state pH maximum of 8.0. Review of the daily log for the previous 10 days indicated the combined chlorine† level had been 10–17 ppm in the pool and 0.8–8.4 ppm in the spa, exceeding the state limit of 0.5 ppm. No remediation steps were documented. As a result of this outbreak investigation, the hotel installed new automated controllers and liquid chlorine feeders to ensure chemical disinfectant levels were kept within regulatory limits.

## Discussion

For almost 100 years, pool chemicals have provided the primary barrier to the transmission of infectious pathogens in treated recreational water venues. However, improper pool chemical handling and storage practices and poor pool operation can cause injuries (3–6), despite their preventable nature. The need to maximize the health benefits of water-based physical activity (7) while minimizing the risk for transmission of infectious pathogens and pool chemical–associated health events should translate into pool owners and operators making prevention of these adverse health events a core element in managing risk at both public and residential treated recreational water venues (Box). With NEISS estimating approximately 4,900 pool chemical–associated injuries for 2012, increased awareness about these injuries and how they can be prevented is needed.

The Minnesota pool chemical–associated health event highlights the need for improvements in training and pool operation. Multiple factors might have contributed to this event. First, chlorine levels and pH documented days after the event exceeded Minnesota’s maximum allowable limits and suggest that the original automated systems to monitor and feed chemicals were not functioning properly. Second, the pool operators either 1) did not check the pool chemistry or equipment as required or 2) identified problems and either did not resolve them or failed to document remediation steps taken. Third, as with almost half of NEISS pool chemical–associated health events, this event occurred during a weekend, a time when pool and spa use might be increased and the likelihood of a trained operator being on duty might be decreased.

BOX **CDC recommendations for prevention of pool chemical–associated injuries for public pool operators and residential pool owners**

**Before you use pool chemicals**

- Get trained in pool chemical safety (e.g., during an operator training course)
- Ask for help if you are not trained for specific tasks
- Read entire product label or Safety Data Sheet (SDS) before using

**Using pool chemicals safely**

- Keep young children away when handling chemicals
- Dress for safety by wearing appropriate safety equipment (e.g., safety goggles, gloves, and respirator)
- Read chemical product label before each use
  – Handle in a well-ventilated area
  – Open one product container at a time and close it before opening another
  – Minimize dust, fumes, and splashes
  – Measure carefully
- Never mix
  – chlorine products with acid; this could create toxic gases
  – different pool chemicals (e.g., different types of chlorine products) with each other or with any other substance
- Only predissolve pool chemicals when directed by product label
  – If product label directs predissolving, add pool chemical to water; never add water to pool chemical because a violent (potentially explosive) reaction can occur

Additional information on pool chemical safety is available at http://www.cdc.gov/healthywater/swimming/pools/preventing-pool-chemical-injuries.html.

The findings in this report are subject to at least four limitations. First, although NEISS data provide a snapshot of pool chemical–associated injuries leading to ED visits, they do not characterize the epidemiology of pool chemical–associated injuries that do not result in an ED visit. Second, missing NEISS data limits understanding of basic characteristics of these adverse health events (e.g., patient’s race) and appropriate points for intervention (e.g., public versus residential settings). Third, a few of the events could have been misclassified as being caused by pool chemicals when they were not (e.g., dermatitis caused by *Pseudomonas* rather than pool chemicals). Finally, water chemistry can change quickly, making it difficult to determine the etiology of and factors contributing to a pool chemical–associated health event.

The continuing occurrence of pool chemical–associated health events and drowning in pools (9,10), as well as the significantly increased annual incidence of recreational water–associated outbreaks (range = 6–84 outbreaks) during 1978–2010 (which primarily is associated with treated recreational water venues and caused by the extremely chlorine-tolerant *Cryptosporidium* [8]), underscore the need for regulators at the state and local levels to optimize protection of swimmer and aquatics staff health, in part, by regularly updating state and local codes for public treated recreational water venues. This updating process requires staffing, resources, and expertise that might not always be available to individual jurisdictions. Consequently, CDC has been leading a national consortium of public health, aquatics sector, and academic stakeholders to develop model guidance (i.e., the MAHC [1]) to aid state and local agencies in incorporating the latest science and best practices into their codes covering design and construction, operation and maintenance, and policies and management of public treated recreational water venues. The first edition of the MAHC will be posted in summer 2014 after the last of two public comment periods closes May 27, 2014. The MAHC will be periodically updated based on the latest reported data in peer-reviewed scientific journals, changes occurring in the aquatics sector (e.g., development of new treated recreational water venue types), and stakeholder input. Areas of the MAHC that should assist in decreasing the incidence of pool chemical–associated health events include requiring operator training, which covers pool chemical safety (e.g., wearing personal protective equipment while handling pool chemicals), and engineering changes to prevent incompatible pool chemicals from mixing.

What is already known on this topic?

Chemicals are added to treated recreational water venues (e.g., pools, hot tubs/spas, and interactive fountains) to inactivate pathogens and maximize the efficacy of the disinfection process by controlling pH. However, these chemicals can cause injuries when handled or stored improperly. Pool chemical–associated health events are preventable.

What is added by this report?

In 2012, an estimated total of approximately 4,900 persons visited an emergency department for pool chemical–associated injuries. Almost half of the patients (46.9%) were aged <18 years. More than a third (36.1%) of the injuries occurred at a residence.

What are the implications for public health practice?

CDC’s Model Aquatic Health Code (available at http://www.cdc.gov/mahc) is a resource that state and local agencies can use to optimize prevention of injuries and illness associated with public treated recreational water venues.

## Figures and Tables

**FIGURE:**
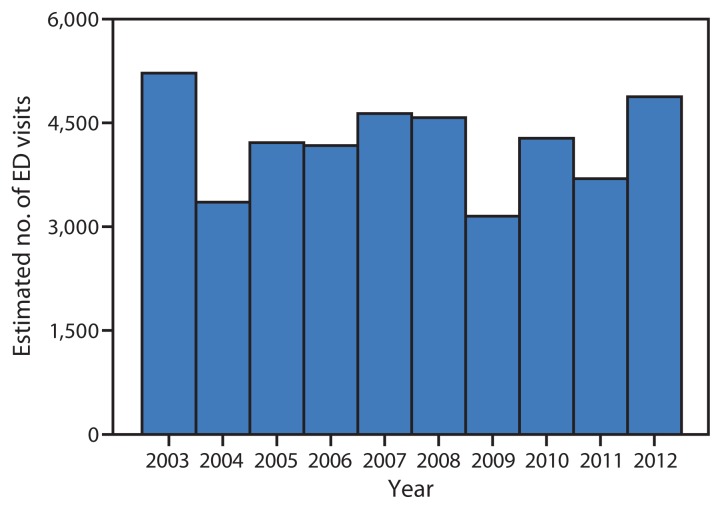
Estimated number of emergency department (ED) visits for injuries associated with pool chemicals — United States, National Electronic Injury Surveillance System, 2003–2012

**TABLE:** Estimated number, percentage, and rate of pool chemical–associated injuries treated in emergency departments — United States, National Electronic Injury Surveillance System (NEISS), 2012

Characteristic	Actual count	Weighted estimate*†	95% CI	%§	Annual rate¶
**Total**	**109**	**4,876**	**(2,821–6,930)**	**100.0**	**1.6**
**Injury diagnosis**					
Poisoning**	50	2,167	(1,219–3,116)	44.5	0.7
Dermatitis/Conjunctivitis	33	1,581	(385–2,778)	32.4	—
Chemical burns	9	469	(16–922)	9.6	—
Other	17	657	(234–1,081)	13.5	—
**Affected body part**					
All parts of the body (>50% of body)††	55	2,218	(1,269–3,167)	45.5	0.7
Eyeball	34	1,525	(572–2,478)	31.3	—
Other (e.g., upper trunk [not shoulder], hand, or foot)	20	1,133	(419–1,847)	23.2	—
**Patient disposition**					
Treated and released (or examined and released) without treatment	101	4,394	(2,804–5,983)	90.1	1.4
Treated and admitted for hospitalization (within same facility)	6	332	(0–701)	6.8	—
Treated and transferred to another hospital	1	79	(0–240)	1.6	—
Held for observation (includes admitted for observation)	1	71	(0–214)	1.5	—
**Incident location**					
Residence	40	1,759	(718–2,799)	36.1	—
Place of recreation or sports	10	408	(32–784)	8.4	—
School	1	70	(0–212)	1.4	—
Other public property	13	641	(0–1,380)	13.1	—
Unknown	45	1,998	(1,057–2,940)	41	—
**Patient age (yrs)**					
0–17	53	2,289	(965–3,613)	46.9	3.1
18–45	23	850	(421–1,278)	17.4	0.7
46–64	28	1,518	(811–2,225)	31.1	1.9
≥65	5	218	(0–441)	4.5	—
**Patient sex**					
Male	72	3,144	(1,832–4,456)	64.5	2.0
Female	37	1,731	(894–2,569)	35.5	1.1
**Patient race/ethnicity**					
White, non-Hispanic	66	3,468	(2,536–4,401)	71.1	—
Hispanic	7	443	(0–1,062)	9.1	—
Black, non-Hispanic	14	309	(69–549)	6.3	—
Other (e.g., multiple race)	1	6	(0–18)	0.1	—
Unknown	21	649	(34–1,264)	13.3	—

**Abbreviation:** CI = confidence interval.

* Each case was weighted based on the inverse probability of the hospital being selected, and the weights were summed to produce national estimates.

† Categorical counts might not total 4,876 because of rounding.

§ Categorical percentages might not total 100% because of rounding.

¶ Rates per 100,000 person-years were calculated using U.S. Census Bureau population estimates (available at http://www.census.gov/popest/data); 95% CIs were calculated using SAS survey procedures that accounted for the sample weights and complex sampling design. If the sample count was <20 or the coefficient of variation >30%, the estimate was considered unstable and not reported. Rates by incident location and race/ethnicity are not reported because of the high percentage of missing data.

** Poisoning includes ingestion as well as inhalation of vapors, fumes, or gases.

†† For a poisoning injury diagnosis, NEISS requires that affected body part be coded as “all parts of the body (>50% of body).”

## Footnotes

* Chlorine in water (found as an aqueous mixture of hypochlorous acid and hypochlorite anion) that can serve as an effective disinfectant (also referred to as free available chlorine or residual chlorine).

† Chlorine that has reacted with organic or inorganic compounds in the water is no longer an effective disinfectant, and might cause ocular and respiratory irritation.
